# Case Report: Myelin oligodendrocyte glycoprotein antibody–associated cortical encephalitis masked by peri-ictal magnetic resonance imaging abnormalities

**DOI:** 10.3389/fimmu.2026.1890365

**Published:** 2026-08-21

**Authors:** Koki Yoshizawa, Atsuhiko Sugiyama, Yuki Nakagawa, Masahiro Namiki, Tomoki Suichi, Masahiro Mori

**Affiliations:** Department of Neurology, Graduate School of Medicine, Chiba University, Chiba, Japan

**Keywords:** autoimmune epilepsy, fluid-attenuated inversion recovery-hyperintense lesions in anti-MOG-associated encephalitis with seizures, myelin oligodendrocyte glycoprotein (MOG) antibody–associated disease, peri-ictal magnetic resonance imaging abnormalities, status epilepticus

## Abstract

One phenotype of cerebral cortical encephalitis in myelin oligodendrocyte glycoprotein (MOG) antibody–associated disease (MOGAD) is FLAMES (fluid-attenuated inversion recovery-hyperintense lesions in anti-MOG-associated encephalitis with seizures). FLAMES is characterized by its imaging features and epileptic seizures. We present a case in which these findings were masked by peri-ictal magnetic resonance imaging abnormalities (PMAs) associated with status epilepticus, making differentiation difficult. A middle-aged woman presenting with persistent right upper limb weakness and aphasia was admitted to our hospital. The patient was diagnosed with nonconvulsive status epilepticus based on electroencephalogram findings. Brain magnetic resonance imaging revealed findings consistent with typical PMAs. Although the seizures’ underlying etiology could not be identified initially, testing confirmed the presence of MOG immunoglobulin G in serum and cerebrospinal fluid, leading to a definitive diagnosis of FLAMES. FLAMES and PMAs share significant radiological overlap. Clinicians should maintain a high index of suspicion for MOGAD in middle-aged patients presenting with new-onset epilepsy of unknown origin, even when radiological findings appear to be typical PMAs.

## 1 Introduction

Myelin oligodendrocyte glycoprotein (MOG) antibody–associated disease (MOGAD) is a demyelinating disease of the central nervous system characterized by MOG immunoglobulin G (MOG-IgG) positivity (1). Since unilateral cortical encephalitis was first described by Ogawa et al. (2), cortical encephalitis has been increasingly recognized as a clinical manifestation of MOGAD. Budhram et al. proposed the term FLAMES (fluid-attenuated inversion recovery-hyperintense lesions in anti-MOG-associated encephalitis with seizures) for a rare phenotype of cortical encephalitis with seizures in MOGAD based on its clinical and radiological features (3). FLAMES typically presents as cortical fluid-attenuated inversion recovery (FLAIR) hyperintensity on magnetic resonance imaging (MRI) (3). However, status epilepticus induces metabolic changes and insufficient compensatory mechanisms, leading to cytotoxic and vasogenic edema (4). The resulting peri-ictal MRI abnormalities (PMAs) associated with status epilepticus can also exhibit cortical FLAIR hyperintensity and often resemble the radiological features of FLAMES, making them difficult to distinguish (3). The characteristic MRI findings of FLAMES may be modified or obscured by PMAs when status epilepticus is superimposed on FLAMES. Herein, we report a case of FLAMES, wherein the characteristic imaging findings were masked by typical PMAs.

## 2 Case description

A 50-year-old right-handed woman was admitted to our hospital with a history of transient right-hand weakness 15 days prior, generalized tonic stiffening 11 days prior, and persistent aphasia and right-hand clumsiness that had begun 7 days before admission. The patient had an unremarkable medical history except for an overactive bladder. There was no family history of autoimmune diseases or epilepsy.

**Figure 1:**
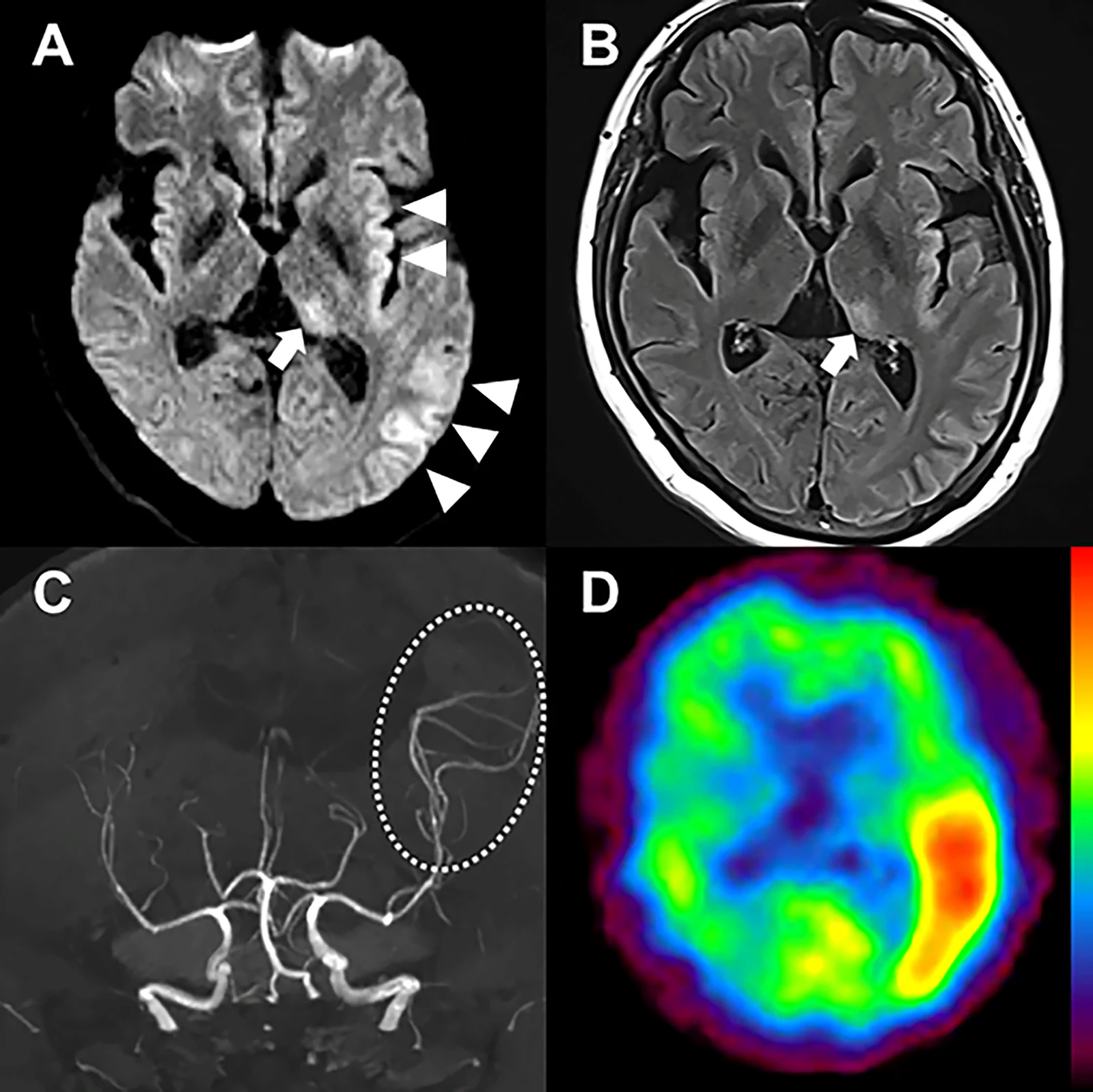
Imaging findings on the first admission. **(A)** Diffusion-weighted imaging (DWI) shows hyperintensity in the left cerebral cortex (white arrow heads) and left pulvinar of the thalamus (white arrows). **(B)** Fluid-attenuated inversion recovery imaging presents hyperintensity in the same regions as seen on DWI (white arrow) and hypointensity in the subcortical white matter in the left temporal lobe. **(C)** Magnetic resonance angiography reveals increased vascularity in the left middle cerebral artery territory (white dotted line). **(D)** Iodine-123-labeled N-isopropyl-p-iodo-amphetamine single-photon emission computed tomography shows hyperperfusion in the left temporal and parietal lobes.

**Figure 2:**
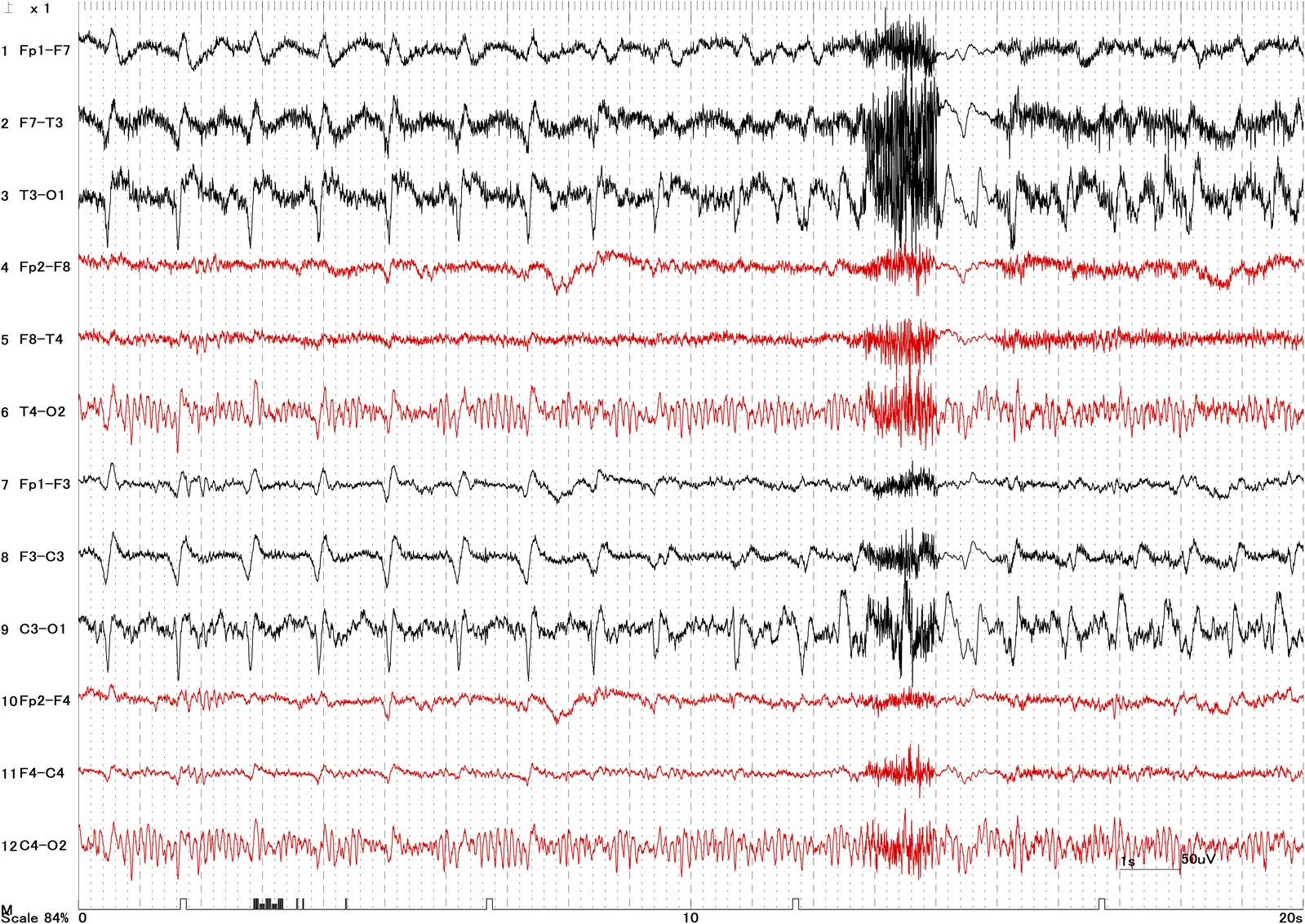
Electroencephalogram shows 1 Hz lateralized periodic discharges maximal over the left occipital area in anterior–posterior bipolar montage (sensitivity: 10 μV/mm, high-frequency filter: 60 Hz, time constant: 0.3 s).

On admission, the patient was conscious and alert but had motor aphasia. Moreover, the patient had right-hand paresis. No convulsions were observed. Blood tests on admission were almost unremarkable. However, serum anti-cyclic citrullinated peptide and anti-glutamic acid decarboxylase (GAD) antibodies were elevated [5.0 U/mL (reference range: <4.5 U/mL) and 17.0 U/mL (reference range: <5.0 U/mL), respectively]. Cerebrospinal fluid (CSF) analysis showed an increased number of white blood cells (13 cells/µL), all of which were mononuclear. However, CSF protein and glucose levels were normal, and CSF cultures were negative. Polymerase chain reaction assays for varicella zoster virus and herpes simplex virus in CSF were below the detection limit. Conversely, oligoclonal bands (OCBs) were positive. Brain MRI performed at another hospital 4 days before admission revealed hyperintensity along the left cerebral cortex in the parietal, temporal, and insular lobes on diffusion-weighted imaging (DWI) (**Figure 1A**) and iso- or hypointensity in the same region on apparent diffusion coefficient mapping. Lesions in the left cerebral cortex appeared hyperintense and edematous on FLAIR imaging, whereas the subcortical white matter showed hypointensity (**Figure 1B**). No contrast enhancement was observed on post-contrast T1-weighted imaging. The left pulvinar of the thalamus also appeared hyperintense on DWI and FLAIR imaging but isointense on apparent diffusion coefficient mapping. No abnormal signal was observed in the left hippocampus. Increased vascularity was observed in the left middle cerebral artery on magnetic resonance angiography (MRA) (**Figure 1C**). Iodine-123-labeled N-isopropyl-p-iodo-amphetamine single-photon emission computed tomography performed on day 2 of admission showed increased uptake in the left temporal and parietal lobes (**Figure 1D**). No increased uptake was observed in the left pulvinar of the thalamus. Initial electroencephalogram (EEG) on the day of admission revealed lateralized periodic discharges (LPDs) mainly in the left occipital region (**Figure 2**). The frequency of these LPDs evolved from 0.5 Hz to 1.5 Hz. Moreover, their location expanded to the temporal regions, and their morphology changed from blunt to sharp. These LPDs persisted for at least 10 min.

The patient was diagnosed with nonconvulsive status epilepticus, and intravenous levetiracetam and lacosamide were administered as antiseizure medications (ASMs). Because of the presence of pleocytosis, acyclovir was administered until herpes simplex encephalitis was ruled out. The Aphasia Quotient of the Western Aphasia Battery performed on day 15 of treatment was 70.8, suggesting motor aphasia and agraphia. Repeated brain MRI performed 35 days after the initial examination revealed the disappearance of abnormal signal changes on DWI and an improvement in high intensity in the pulvinar of the thalamus and the cerebral cortex on FLAIR imaging. Follow-up EEG performed on day 3 of treatment showed intermittent theta and delta activity in the left occipital region, but LPDs had disappeared. Right upper limb paresis and motor aphasia were improving but persisted, and at this point were considered a postictal state. The right upper limb paresis resolved on day 11 of treatment, but mild motor aphasia remained and was considered a sequela of severe status epilepticus. We investigated the etiology of new-onset seizures. Central nervous system infection was ruled out based on the results of viral DNA testing and culture studies of the CSF. Although serum anti-GAD and anti-cyclic citrullinated peptide antibodies were positive, their low titers and atypical clinical findings made GAD antibody–associated encephalitis and rheumatoid meningitis unlikely. An autoimmune etiology was initially considered unlikely as the patient’s symptoms and examination abnormalities improved by administering only ASMs. Following rehabilitation, the patient was monitored as an outpatient. No new MRI abnormalities (e.g., a tumor) appeared over the following 10 months.

A few days after reducing the dose of levetiracetam because of seizure stability and side effects of somnolence, the patient was admitted to our hospital following a tonic-clonic seizure, persistent total aphasia, and right-hand weakness. Upon admission, the patient was unable to follow commands and exhibited myoclonus and right-hand paresis. The patient’s Aphasia Quotient of the Western Aphasia Battery was 53.3, suggesting total aphasia.

**Figure 3:**
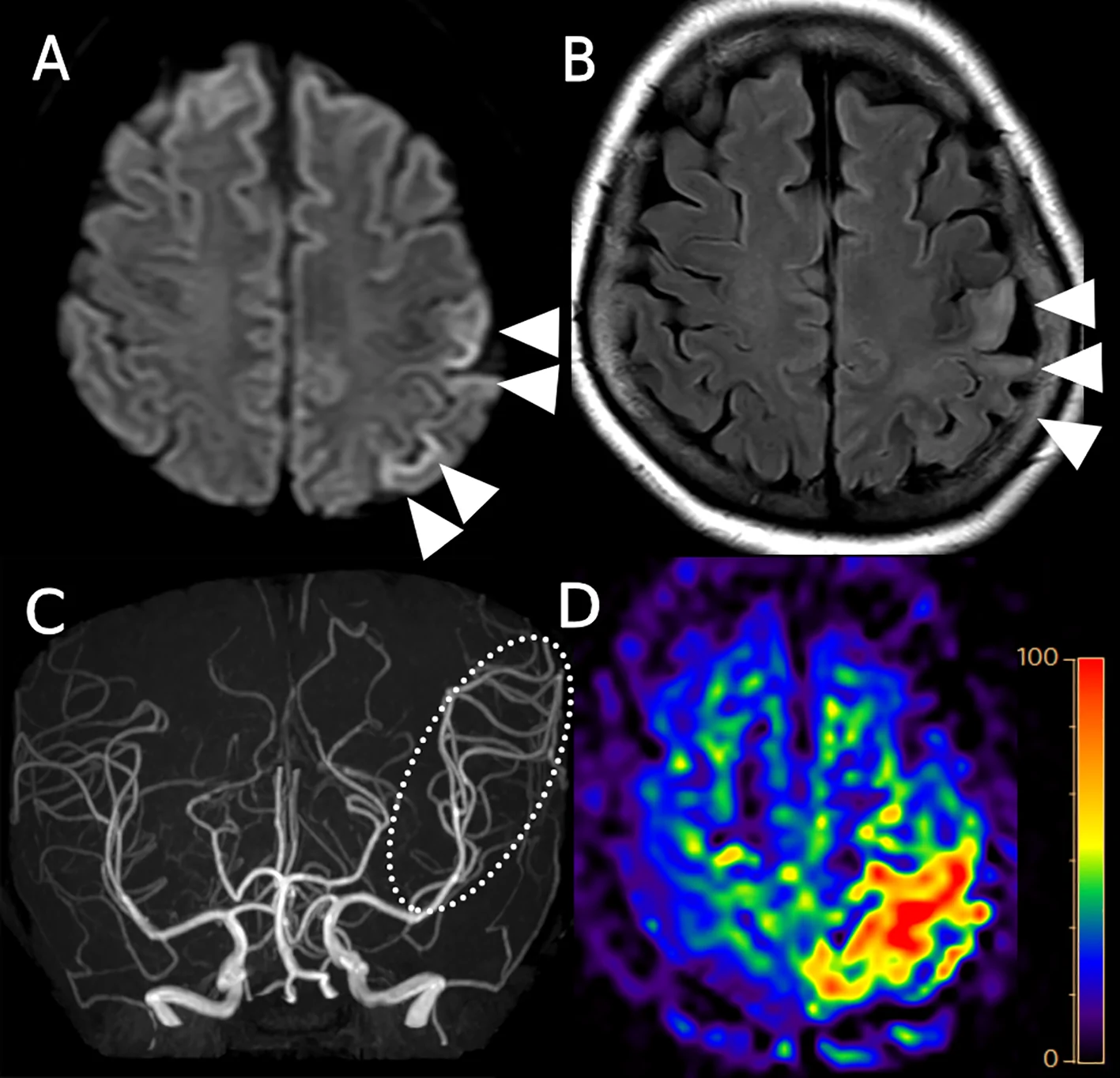
Magnetic resonance imaging findings on the second admission. **(A)** Diffusion-weighted imaging (DWI) shows hyperintensity along the left parietal cortex (white arrowheads). **(B)** Fluid-attenuated inversion recovery imaging demonstrates hyperintensity in the same region as observed on DWI (white arrowheads). **(C)** Magnetic resonance angiography reveals increased vascularity in the left middle cerebral artery territory (white dotted line). **(D)** Arterial spin labeling demonstrates hyperperfusion in the left parietal lobe.

Serum anti-GAD and anti-cyclic citrullinated peptide antibodies, which were slightly elevated during the previous admission, were negative. CSF analysis showed no pleocytosis, and protein and glucose levels were within normal limits. Brain MRI revealed hyperintensity along the left parietal cortex on DWI (**Figure 3A**), and the same parietal cortex and subcortical white matter appeared hyperintense on FLAIR imaging (**Figure 3B**). Meanwhile, MRA and arterial spin labeling demonstrated hyperperfusion in the left hemisphere (**Figures 3C, D**). EEG revealed 1–2 Hz LPDs localized to the left parietal region. No myoclonus was observed during the EEG recording, and therefore, a time-locked relationship with the LPDs could not be confirmed. This rhythmic pattern disappeared following diazepam administration.

The patient was diagnosed with nonconvulsive status epilepticus and administered with intravenous levetiracetam and fosphenytoin. Thereafter, the patient’s myoclonus, right upper limb paresis, and aphasia improved. On follow-up EEG, the rhythmic patterns had resolved. The dose of lacosamide was increased to prevent seizure recurrence.

**Figure 4:**
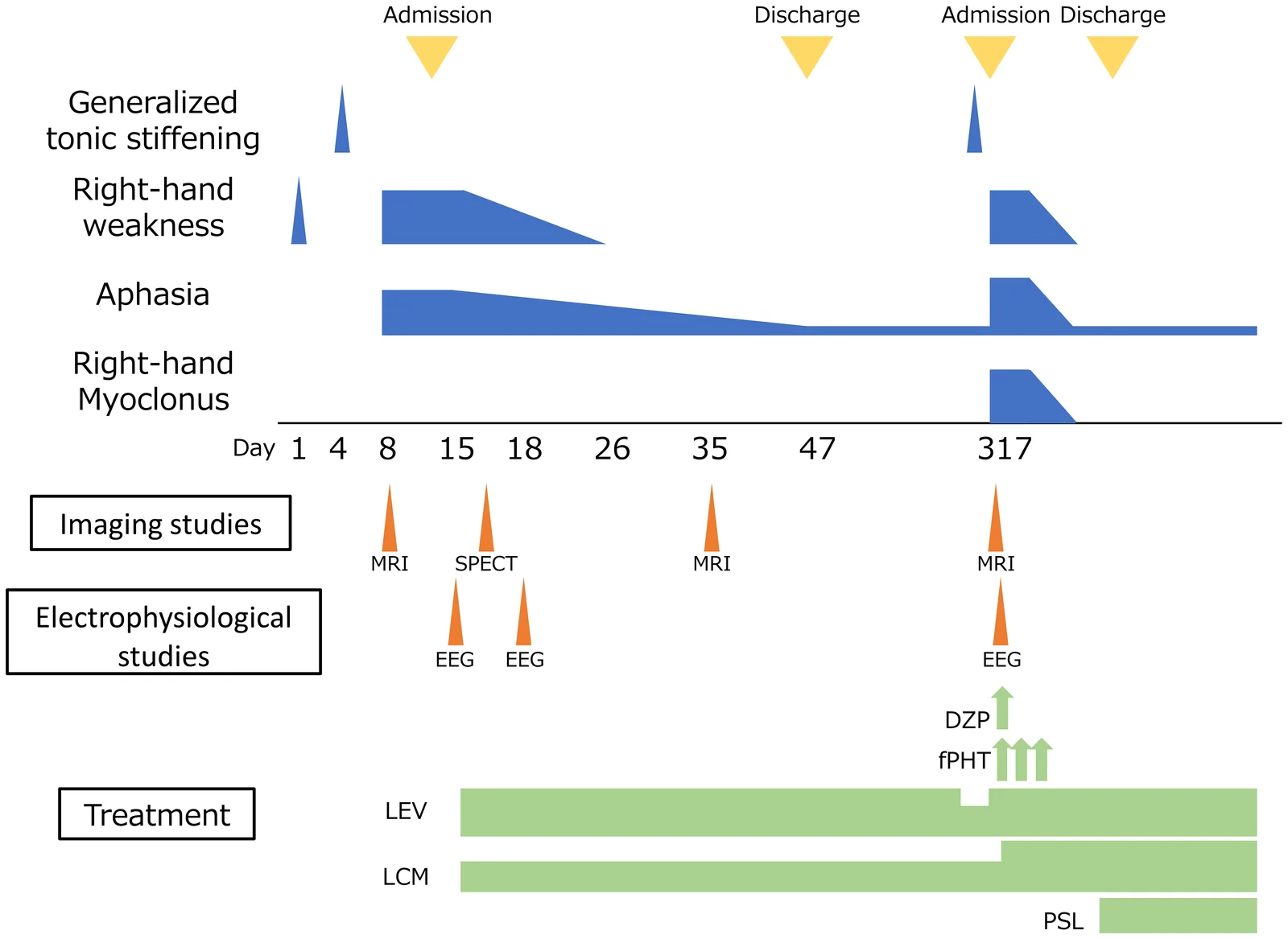
Patient’s clinical timeline. This figure illustrates the clinical symptoms and timing of imaging studies, electrophysiological studies, and treatment. DZP, diazepam; EEG, electroencephalography; fPHT, fosphenytoin; LCM, lacosamide; LEV, levetiracetam; MRI, magnetic resonance imaging; PSL, prednisolone; SPECT, single-photon emission computed tomography.

The absence of tumors on follow-up MRI and the prompt recurrence of seizures during ASM tapering suggested an autoimmune etiology as the underlying mechanism of the patient’s epilepsy, especially considering the previously noted pleocytosis and positive OCBs. Subsequent testing by cell-based assay for MOG-IgG in stored serum and CSF samples from the first seizures demonstrated positive titers of 1:64 and 1:16, respectively. Anti-metabotropic glutamate receptor 1 antibodies in the CSF were negative. The patient was diagnosed with FLAMES, and oral prednisolone was initiated at 0.5 mg/kg/day to prevent relapses. The symptoms returned to the baseline level observed prior to the second admission, and the patient was subsequently discharged (**Figure 4**). The patient expressed relief upon learning the definitive diagnosis and understanding the reason for her recurrent symptoms.

## 3 Discussion

This case describes a patient in whom the imaging findings of FLAMES were obscured by typical PMAs associated with status epilepticus, complicating diagnosis and treatment. Several cohort studies have reported FLAMES presenting with status epilepticus (5, 6), and prior reports have noted imaging similarities between FLAMES and PMAs, including cortical hyperintensity on T2-FLAIR (3, 5). However, there are no reports of adult MOGAD cases in which PMA and FLAMES imaging findings clearly overlapped.

In this case, PMA-related findings predominated, masking the characteristic features of FLAMES. Notably, FLAMES typically presents with unilateral cortical T2-FLAIR hyperintensity, sulcal FLAIR hyperintensity, leptomeningeal enhancement, increased vascularity on MRA, and hyperperfusion on perfusion imaging (2, 7–9). Conversely, PMAs commonly show cortical diffusion restriction and T2-FLAIR hyperintensity, along with hyperperfusion. Diffusion restriction in the hippocampus and pulvinar is also characteristic of PMAs (4, 10). Notably, pulvinar lesions observed in our case have not been reported in adult FLAMES and are rare even in pediatric cases (11, 12). By contrast, such lesions are reported in approximately 20% of patients with status epilepticus and show high specificity (10), supporting PMAs as the primary process. Conversely, sulcal FLAIR hyperintensity and leptomeningeal enhancement are relatively characteristic of FLAMES and should raise suspicion when present; however, these were absent, and most imaging findings were consistent with PMAs.

Subcortical T2-FLAIR hypointensity observed in this case is frequently reported in FLAMES (13) and is noteworthy. Although not entirely specific—similar findings may occur in seizures associated with nonketotic hyperglycemic hyperosmolar state (14)—such situations are limited. Therefore, this imaging finding may help differentiate PMAs from FLAMES (**Table 1**).

**Table 1:** Comparison of imaging features between FLAMES and PMAs, including findings from the present case.

Imaging feature	FLAMES	PMAs	Present case
Distribution	Predominantly unilateral; occasionally bilateral	Variable; follows the seizure focus	Unilateral, left hemisphere
Cortical FLAIR hyperintensity	Common	Common	Present
Cortical DWI hyperintensity	Rare	Common	Present
Restricted diffusion (ADC decrease)	Rare	Common	Present in the cortex; pulvinar isointense on ADC
Sulcal FLAIR hyperintensity	Common	Rare	Absent
Leptomeningeal enhancement	Common	Rare	Absent
Pulvinar involvement	Rare	Common	Present
Hippocampal/medial temporal signal change	Rare	Common	Absent
Subcortical white matter FLAIR hypointensity	Frequent	Associated with the underlying disease	Present
Hyperperfusion on ASL/SPECT	Common	Common	Present

Frequency terms (common, frequent, and rare) are used as qualitative descriptors and should not be interpreted as exact frequency estimates. ADC, apparent diffusion coefficient; ASL, arterial spin labeling; DWI, diffusion-weighted imaging; FLAIR, fluid-attenuated inversion recovery; FLAMES, fluid-attenuated inversion recovery-hyperintense lesions in anti-MOG-associated encephalitis with seizures; PMAs, peri-ictal MRI abnormalities; SPECT, single-photon emission computed tomography.

The diagnosis of FLAMES is challenging when there are no characteristic imaging findings and clinical symptoms. During initial admission, the Response to Immunotherapy in Epilepsy and Antibody Prevalence in Epilepsy and Encephalopathy scores (15, 16) were both 3, not strongly suggesting an autoimmune etiology. FLAMES typically presents with encephalitic symptoms such as altered mental status, headache, or fever in addition to seizures (6, 17), but these were absent initially. Consequently, testing for autoantibodies, including MOG-IgG, was delayed. An autoimmune mechanism was also considered unlikely because symptoms improved with ASMs alone.

In retrospect, several red flags were present. CSF analysis revealed significant pleocytosis and OCB positivity. In MOGAD, pleocytosis (>5 white blood cells/mm^3^) is a frequent finding, reported in >50% of MOGAD cases (18) and in nearly all FLAMES cases (17). Although OCB positivity is more commonly associated with multiple sclerosis, it is reported in approximately 15% of MOGAD cases (19). While pleocytosis and OCB positivity have been described in status epilepticus (20, 21), they are less common than in encephalitis. Therefore, in patients with new-onset seizures and CSF pleocytosis or OCB positivity, testing for MOG-IgG should be considered.

It is critical to differentiate symptoms caused by epileptic seizures from those due to encephalitis. FLAMES frequently presents with focal neurological deficits (6), whereas nonconvulsive status epilepticus (NCSE) and postictal states can produce similar deficits. In the present case, although continuous EEG monitoring was unavailable, epileptiform discharges had resolved by day 3 of treatment. However, motor aphasia and right upper limb paresis persisted and were initially considered postictal. Postictal deficits usually resolve within 24 hours; even Todd’s paresis typically has a median duration of 2.5 days (22). The prolonged deficits in this case suggest focal cortical dysfunction due to encephalitis. Therefore, a mismatch between EEG findings and clinical symptoms, as well as prolonged symptom persistence after seizure cessation, can serve as red flags suggesting underlying FLAMES. This is especially true in the absence of prominent fever or altered mental status. Ultimately, although FLAMES is defined by its imaging features and epileptic seizures, the diagnosis should be established within the comprehensive context of the clinical course, CSF, and EEG findings, rather than based solely on imaging.

Clinicians should consider testing for MOG-IgG in adult patients with new-onset seizures who present with these red flags. Although the Antibody Prevalence in Epilepsy and Encephalopathy score is useful for predicting autoantibody positivity in patients with acute or subacute onset epilepsy (16), it does not include MOG-IgG. Current criteria recommend testing when a clinical MOGAD phenotype is present (1), but cerebral cortical encephalitis and FLAMES are relatively uncommon (1). Diagnostic delay is likely when typical MRI findings are obscured by PMAs or when encephalitic symptoms are absent, as in this case. Notably, key red flags include subcortical T2-FLAIR hypointensity, CSF pleocytosis, and discordance between EEG and clinical findings.

Interpretation of autoantibody testing also requires careful consideration. Up to 28% of MOG-IgG-positive results may be false positives, particularly at low titers (23). In isolated new-onset seizures without additional symptoms, autoimmune encephalitis is unlikely (24), and MOG-IgG testing is generally not recommended. In the present case, GAD antibodies were weakly positive during the initial attack. However, GAD-associated encephalitis typically presents with temporal lobe epilepsy or limbic encephalitis (25), which did not match this phenotype. Furthermore, clinically significant cases usually show high antibody titers, whereas low-titer positivity may occur in healthy individuals (25). Therefore, GAD-associated encephalitis was considered unlikely.

Accurate and early diagnosis of MOGAD is crucial, as delayed treatment worsens prognosis. High-dose corticosteroids are recommended as first-line acute therapy (26), and delayed treatment has been associated with increased relapse risk and a reduced rate of seronegative conversion (27). Early immunotherapy is also an independent prognostic factor in autoimmune encephalitis (28). In this case, diagnostic delay due to overlapping imaging findings precluded early immunotherapy, which may have contributed to epileptogenesis and persistent aphasia. This underscores the importance of early recognition and treatment.

In conclusion, FLAMES and PMAs share many imaging similarities and can clinically overlap. MOG antibody testing should be considered even when MRI findings mimic typical PMAs in middle-aged patients presenting with new-onset epilepsy of unknown etiology, especially if clinical red flags (e.g., pleocytosis or OCB positivity) are present.

## Data Availability

The original contributions presented in the study are included in the article/supplementary material. Further inquiries can be directed to the corresponding author.
